# Endothelial Insulin Receptor Restoration Rescues Vascular Function in Male Insulin Receptor Haploinsufficient Mice

**DOI:** 10.1210/en.2018-00215

**Published:** 2018-05-15

**Authors:** Anshuman Sengupta, Peysh A Patel, Nadira Y Yuldasheva, Romana S Mughal, Stacey Galloway, Hema Viswambharan, Andrew M N Walker, Amir Aziz, Jessica Smith, Noman Ali, Ben N Mercer, Helen Imrie, Piruthivi Sukumar, Stephen B Wheatcroft, Mark T Kearney, Richard M Cubbon

**Affiliations:** Leeds Institute of Cardiovascular and Metabolic Medicine, LIGHT Laboratories, The University of Leeds, Leeds, United Kingdom

## Abstract

Reduced systemic insulin signaling promotes endothelial dysfunction and diminished endogenous vascular repair. We investigated whether restoration of endothelial insulin receptor expression could rescue this phenotype. Insulin receptor knockout (IRKO) mice were crossed with mice expressing a human insulin receptor endothelial cell–specific overexpression (hIRECO) to produce IRKO-hIRECO progeny. No metabolic differences were noted between IRKO and IRKO-hIRECO mice in glucose and insulin tolerance tests. In contrast with control IRKO littermates, IRKO-hIRECO mice exhibited normal blood pressure and aortic vasodilatation in response to acetylcholine, comparable to parameters noted in wild type littermates. These phenotypic changes were associated with increased basal- and insulin-stimulated nitric oxide production. IRKO-hIRECO mice also demonstrated normalized endothelial repair after denuding arterial injury, which was associated with rescued endothelial cell migration *in vitro* but not with changes in circulating progenitor populations or culture-derived myeloid angiogenic cells. These data show that restoration of endothelial insulin receptor expression alone is sufficient to prevent the vascular dysfunction caused by systemically reduced insulin signaling.

## Teaser

By normalizing endothelial insulin receptor expression in mice with otherwise global insulin receptor haploinsufficiency, we show rescue of vascular endothelial function and repair.

Many cellular lineages are insulin sensitive, and so insulin signaling is likely to play a critical physiological role beyond regulating systemic glucose homeostasis. Mice with global or endothelium-restricted reduction in insulin signaling exhibit vascular endothelial dysfunction, hypertension, accelerated atherosclerosis, and reduced vascular repair (1–6). We have recently shown that mice with increased endothelial insulin signaling [due to human insulin receptor endothelial cell–specific overexpression (hIRECO)] also have endothelial dysfunction and accelerated atherosclerosis, further supporting the need for normal endothelial insulin signaling in vascular homeostasis (7). However, it remains unclear whether normalization of endothelial insulin receptor expression in mice with systemically reduced insulin receptor expression is sufficient to rescue their vascular function. This would more convincingly prove the role of endothelial insulin receptors, because gene knockout studies can report phenotypes due to off-target “compensatory” gene expression rather than loss of the target gene (8). In other words, robust proof of a gene’s function requires phenotypic rescue when gene expression is restored in a knockout model; however, this level of proof is rarely reported by *in vivo* studies.

We have previously demonstrated that mice with global haploinsufficiency of the insulin receptor [insulin receptor knockout (IRKO) mice] are normoglycemic yet have endothelial dysfunction and impaired endothelial repair (3, 4). To establish whether diminished endothelial insulin receptor expression *per se* causes this phenotype, we crossed IRKO mice with hIRECO mice to generate IRKO-hIRECO progeny and contrasted their vascular function with that of IRKO mice; data describing wild-type (WT) littermates are also provided for reference (7).

## Materials and Methods

### Breeding and maintenance of mice

IRKO-hIRECO mice were derived by breeding male IRKO with female hIRECO mice (3, 7), resulting in either WT, IRKO, hIRECO, or IRKO-hIRECO progeny; hIRECO littermates were not used in this study. Briefly, IRKO mice have global haploinsufficiency of the insulin receptor, whereas hIRECO mice express a human insulin receptor transgene under transcriptional control of the Tie2 promoter-enhancer. The absence of hIRECO transgene expression in myeloid cells has been confirmed in our previous work (7). Mice were housed in a conventional animal facility with a 12-hour light/dark cycle and received *ad libitum* access to a standard laboratory diet unless fasted for metabolic testing. Male mice aged 3 to 4 months were used, except for pulmonary endothelial cell isolation, which used 4- to 6-week-old male mice. Genotyping was performed with PCR and genomic DNA derived from ear notches (3, 7). Primer sequences are listed in Table 1. All procedures were performed according to accepted standards of humane animal care, approved by the ethical review committee of the University of Leeds, and conducted under license from the United Kingdom Home Office.

**Table 1.** Details of All Quantitative PCR Primers

Primer	Sequence or Manufacturer Code
Genotyping	
IRKO (forward 1)	TTAAGGGCCAGCTCATTCCTCC
IRKO (forward 2)	AGCTGTGCACTTCCCTGCTCAC
IRKO (reverse)	TCTTTGCCTGTGCTCCACTCTCA
hIRECO (forward)	ACGTCAGTAGTCATAGGAACTG-CGGTCG
hIRECO (reverse)	TGCCTTGATTCACCAGATGCTG-AGG
Quantitative RT-PCR	
Human insulin receptor (forward)	TGCCACCAACCCCTCTGT
Human insulin receptor (reverse)	CGGAGGGTGGTTTCCACTT
Murine *β*-actin (forward), for normalization of human insulin receptor	CGTGAAAAGATGACCCAGATCA
Murine *β*-actin (reverse), for normalization of human insulin receptor	TGGTACGACCAGAGGCATACAG
Murine insulin receptor	TaqMan probe (Thermo Fisher, Waltham, MA) Mm_01211875_m1
Murine *β*-actin, for normalization of murine insulin receptor	TaqMan probe (Thermo Fisher) Mm_00607939

### Metabolic assessment

Serum insulin ELISA (Crystal Chem, Zaandam, Netherlands), glucose tolerance tests and insulin tolerance tests were performed as previously described in 3- to 4-month-old mice (9).

### Blood pressure assessment

Blood pressure was measured by tail volume-pressure recording in conscious 3- to 4-month-old mice as previously described (3). In brief, tail cuff plethysmography (Coda noninvasive tail blood pressure cuff; Kent Scientific, Torrington, CT) was performed in a restraining chamber heated to 32°C, after mice had undergone two acclimatization sessions on preceding days. During the experimental session, data from the first 10 acclimatization cycles were discarded before three cycles of six blood pressure measurements were collected, the mean of which was used to provide a single data point per mouse. Because our published work has reported only elevated systolic blood pressure in IRKO mice, we measured only systolic blood pressure in these experiments.

### Vascular function studies

Vasomotor function was assessed in aortic rings of 3- to 4-month-old mice, as previously described (9). Rings were mounted in an organ bath containing Krebs-Henseleit buffer (composition, in mmol/L: NaCl 119, KCl 4.7, KH_2_PO_4_ 1.18, NaHCO_3_ 25, MgSO_4_ 1.19, CaCl_2_ 2.5, and glucose 11.0) gassed with 95% O_2_/5% CO_2_. Rings were equilibrated at a resting tension of 3 g for 45 minutes before the experiments. A cumulative dose-response to the constrictor phenylephrine (PE) (1 nmol/L to 100 μmol/L) was first performed. Relaxation responses to cumulative addition of acetylcholine (1 nmol/L to 100 μmol/L) and sodium nitroprusside (SNP) (0.01 nmol/L to 100 μmol/L) were then carried out in rings preconstricted with 300 nmol/L PE. Relaxation responses are expressed as percentage decrement in preconstricted tension. Bioavailable nitric oxide (NO) in aortic segments subjected to isometric tension was measured by recording the change in PE-induced tension elicited by the NO synthase inhibitor L-NMMA (0.1 mmol/L) (7).

### NO synthase activity

Endothelial nitric oxide synthase (eNOS) activity was determined by conversion of [14C]l-arginine to [14C]l-citrulline, as we previously described (9). Aortic rings (5 mm in length) were incubated at 37°C for 20 minutes in HEPES buffer (pH 7.4): 10 mmol/L HEPES, 145 mmol/L NaCl, 5 mmol/L KCl, 1 mmol/L MgSO_4_, 10 mmol/L glucose, and 1.5 mmol/L CaCl_2_, containing 0.25% BSA. We then added 0.5 mCi/mL [14C]l-arginine for 5 minutes, and tissues were stimulated with insulin (100 nmol/L) for 30 minutes before the reaction was stopped with cold PBS, containing 5 mmol/L l-arginine and 4 mmol/L EDTA, after which tissue was denatured in 95% ethanol. After evaporation, the soluble cellular components were dissolved in 20 mmol/L HEPES-Na+ (pH 5.5) and applied to a well-equilibrated DOWEX (Na+ form; Sigma Aldrich, St Louis, MO) column. The [14C]l-citrulline content of the eluate was quantified by liquid scintillation counting and normalized against the weight of tissue used or total cellular protein.

### Vascular injury

Experiments were conducted in 3- to 4-month-old mice as previously described (4). Briefly, femoral arterial injury was produced with an angioplasty guidewire and re-endothelialization assessed 4 days later by infusion of Evans blue dye. Data are presented as the percentage re-endothelialization (absence of Evans blue staining) of a 5-mm arterial segment, 5 mm distal to the aortic bifurcation.

### Flow cytometry

As published (4), saphenous venous blood samples were analyzed with flow cytometry (Becton Dickinson LSR II; BD Biosciences, Franklin Lakes, NJ). After blocking with Fc receptor blocking reagent (BD Biosciences) at a 1:10 dilution (10), Sca-1^+^/Flk-1^+^ and c-Kit^+^ cells were labeled with fluorophore-conjugated primary antibodies (BD Biosciences) at a 1:250 dilution (11–13) and quantified as a percentage of events within the lymphocyte gate, defined by forward-scatter and side-scatter properties. Control specimens, labeled with corresponding fluorophore-conjugated isotype control antibodies (BD Biosciences) at a 1:250 dilution (14–16), were used to define the threshold for antigen expression and to subtract nonspecific fluorescence.

### Cell culture derivation of myeloid angiogenic cells

Mononuclear cells were isolated from blood and spleen, then cultured in EGM-2 medium supplemented with a EGM-2 Bullet Kit (Lonza, Basel, Switzerland) and 20% fetal calf serum; these are identical to the methods used in our previous work to derive cells referred to as angiogenic progenitor cells in our previous publications (4). As per a recent consensus agreement on “endothelial progenitor” nomenclature, we now refer to these as myeloid angiogenic cells, which promote vascular regeneration in a paracrine manner. These cells were enumerated on day 7, based on DiI-acetylated low-density lipoprotein uptake and *Ulex europaeus* lectin–fluorescein isothiocyanate binding (17). The paracrine function of spleen-derived cells was assessed by deriving conditioned medium, as previously described (4). The impact of conditioned medium on human umbilical vein endothelial cell (HUVEC; Promocell, Heidelberg, Germany) scratch wound closure was defined.

### Pulmonary endothelial cell culture

Endothelial cells were isolated from the lungs of 4- to 6-week-old mice; although they were younger than mice used in our other experiments, we found that juvenile lungs produced higher-quality cell preparations for functional studies. As previously described, CD146^+^ pulmonary endothelial cells (PECs) were isolated with immunomagnetic microbeads (Miltenyi Biotec, Bergisch Gladbach, Germany) and cultured in MV2 medium (Promocell), supplemented with 10% fetal calf serum, 100 units/mL penicillin, and 100 mg/mL streptomycin (9). Cells were studied at passage 2 to 3.

### Quantification of endothelial superoxide production

PECs were loaded with 10 mmol/L dihydroethidium and analyzed with a plate reader apparatus (FlexStation; Molecular Devices, San Jose, CA) as previously described (2).

### Endothelial scratch wound assay

A wound was formed in confluent PECs or human umbilical vein endothelial cells with a Woundmaker^TM^ tool (Essen Biosciences, Ann Arbor, MI). Phase contrast images were collected (Olympus CX-41; Olympus Corporation, Tokyo, Japan) immediately and 12 hours later, and the percentage area reduction (wound closure) calculated with ImageJ (National Institutes of Health, Bethesda, MD).

### Endothelial migration assay

As described, PECs were seeded in a Boyden chamber apparatus to define migration toward 50 ng/mL vascular endothelial growth factor (VEGF)-A_165_ (R&D Systems, Minneapolis, MN) (18). The number of migrating cells per microscopic field was counted under standard light microscopy and presented as net migration by subtracting the number of cells migrating in paired control experiments without VEGF-A_165_ gradient.

### Endothelial cell proliferation assay

Subconfluent PECs were exposed to 10 µM 5-ethynyl-2′-deoxyuridine (EdU) in standard growth medium for 4 hours before labeling with Alexa Fluor-488 with a flow cytometry EdU Click-iT Kit (Invitrogen, Carlsbad, CA). The percentage of EdU^+^ cells was defined with flow cytometry (Becton Dickinson LSR II).

### Immunoblotting

As published (7), protein extracts were resolved on 4% to 12% Bis-Tris gels (Invitrogen) and transferred to polyvinylidene fluoride membranes. Blots were probed with the following primary antibodies, with manufacturers and dilutions presented in parentheses: insulin receptor-*β* (Santa Cruz Biotechnology, Dallas, TX; 1:500) (19), *β*-actin (Cell Signaling Technology, Danvers, MA; 1:20,000) (20), eNOS (BD Biosciences, 1:1000) (21), phospho-eNOS S1177 (BD Biosciences, 1:1000) (22), Akt (Cell Signaling Technology, 1:1000) (23), and phospho-Akt S473 (Cell Signaling Technology, 1:2000) (24). Peroxidase-conjugated secondary antibodies (GE Healthcare, Chicago, IL) were then applied at a 1:1000 dilution (25, 26), before increased chemiluminescence (Millipore, Burlington, MA).

### Quantitative RT-PCR Reaction

PECs were lysed in TRI-Reagent (Sigma) and RNA isolated as previously described (7). After reverse transcription (High Capacity cDNA Reverse Transcription Kit; Applied Biosystems, Waltham, MA), quantitative polymerase chain reaction for the human and murine insulin receptor was conducted exactly as published (including primary sequences) (7), and cycle threshold was normalized to *β*-actin using the equation 2^−ΔCT^ × 100. Primer details are presented in Table 1.

### Statistics

Data are presented as mean with SEM in parentheses. Two-sided unpaired Student *t* tests were used for comparisons between IRKO and IRKO-hIRECO mice to test the hypothesis that endothelial normalization of insulin receptor expression would rescue the vascular phenotype of IRKO. WT littermate data are presented solely for reference in key experiments; our published data already show impaired vascular function and repair in IRKO vs WT littermates (3, 4). Statistical significance was defined as *P* < 0.05.

## Results

### IRKO-hIRECO mice have endothelial-specific restoration of insulin receptor expression and signaling

**Figure 1.**
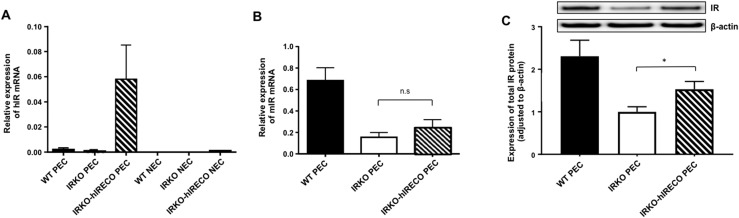
Endothelial insulin receptor expression is normalized in IRKO-hIRECO. (A) Human insulin receptor (hIR) mRNA is detected only in PECs isolated from lungs of IRKO-hIRECO mice and was undetectable in nonendothelial cells (NECs) (n > 4 per group). (B) Murine insulin receptor (mIR) mRNA is comparable in IRKO and IRKO-hIRECO pulmonary endothelial cells, and both are less abundant than in WT littermates (n > 4 per group). (C) Total insulin receptor (IR) protein expression is greater in IRKO-hIRECO than IRKO pulmonary endothelial cells (n = 5, 9, and 13, respectively); representative immunoblots are presented above the panel. Sample sizes are presented in the order WT, IRKO, and IRKO-hIRECO. *P < 0.05. n.s., nonsignificant for IRKO vs IRKO-hIRECO.

Human insulin receptor mRNA was present in IRKO-hIRECO PECs but not in nonendothelial cells from lung preparations (Fig. 1A). Moreover, murine insulin receptor mRNA was comparable in IRKO and IRKO-hIRECO mice, both being significantly lower than in WT littermates (Fig. 1B). Despite testing multiple “species-specific” insulin receptor antibodies, we were unable to find any that unequivocally discriminated between human and mouse isoforms. However, immunoblotting with an antibody reactive to human and murine insulin receptor demonstrated IRKO-hIRECO expression to be intermediate to IRKO and WT (Fig. 1C). Notably, “basal” phosphorylation of Akt and eNOS was reduced in freshly isolated (*i.e.,* noncultured) endothelial cells from nonfasted IRKO-hIRECO vs IRKO mice and was comparable to that of WT controls (Supplemental Fig. 1A and 1B). Because this finding may reflect differences in the *in vivo* exposure of these cells to insulin and other ligands regulating Akt signaling, we also assessed culture-expanded lung endothelial cells (Supplemental Fig. 1C and 1D). Interestingly, these findings suggested that after 4 hours of quiescence in serum-free media and 15 minutes after exposure to 150 nM insulin, Akt phosphorylation was similar in IRKO, IRKO-hIRECO, and WT mice.

### Systemic glucose homeostasis is comparable in IRKO and IRKO-hIRECO mice

**Figure 2.**
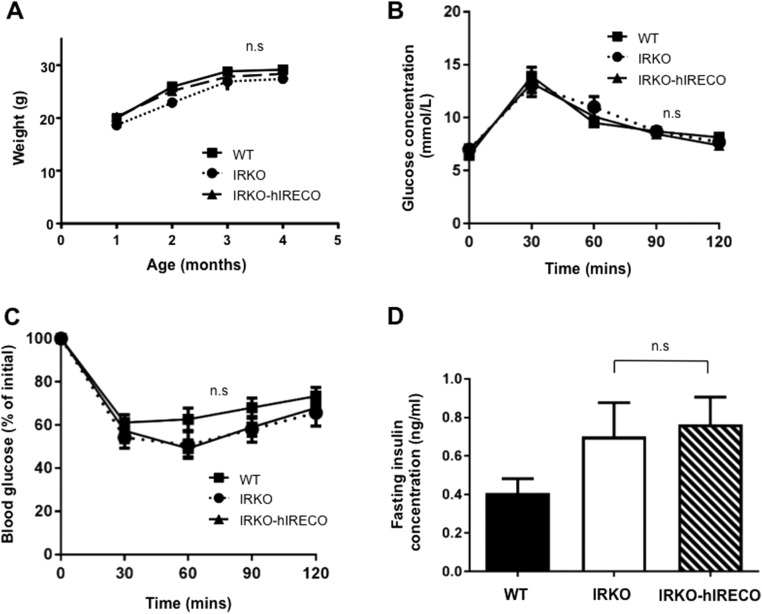
Weight gain and systemic glucose metabolism are unchanged in IRKO-hIRECO mice. (A) Weight gain from weaning to 4 months of age is indistinguishable between IRKO-hIRECO, IRKO, and WT littermates (n > 10 per group). (B) Glucose tolerance was comparable in IRKO-hIRECO and IRKO mice (n = 9, 8, and 7, respectively). (C) Insulin tolerance was comparable in IRKO-hIRECO and IRKO mice (n = 8, 10, and 10, respectively). (D) Serum fasting insulin concentration was comparable in IRKO-hIRECO and IRKO mice (n = 9, 8, and 8, respectively). Sample sizes are presented in the order WT, IRKO, and IRKO-hIRECO. n.s., nonsignificant for IRKO vs IRKO-hIRECO.

Weight gain of IRKO-hIRECO mice was comparable to that of IRKO mice throughout the study period (Fig. 2A). Notably, there was also no difference in glucose or insulin tolerance (Fig. 2B and 2C) or fasting serum insulin concentration (Fig. 2D) between IRKO and IRKO-hIRECO mice. Therefore, any differences in vascular function between IRKO and IRKO-hIRECO mice are unlikely to be related to altered glucose homeostasis. Moreover, no statistically significant differences were noted between WT and IRKO or IRKO-hIRECO mice (Fig. 2A–2D), implying that endothelial insulin receptors are not essential for systemic glucose homeostasis and so may serve alternative roles.

### IRKO-hIRECO mice exhibit rescue of vascular endothelial dysfunction

**Figure 3.**
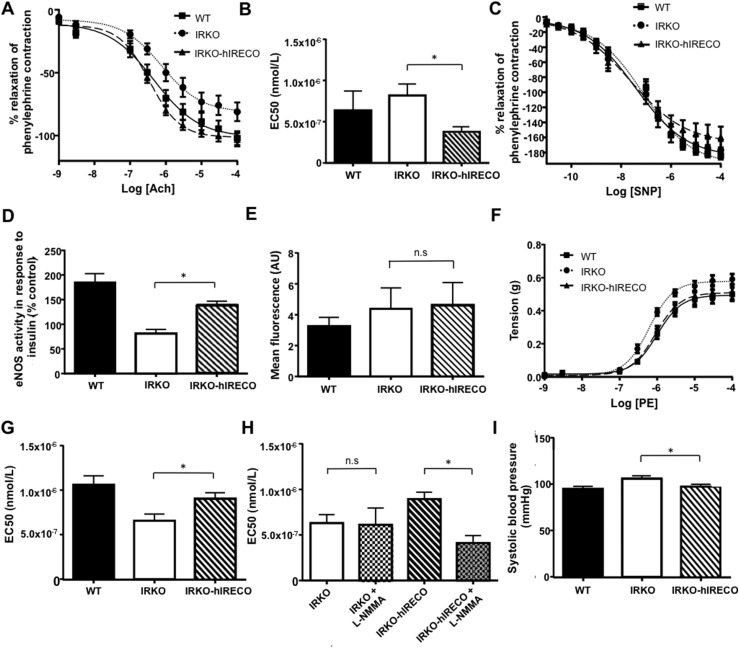
IRKO-hIRECO mice have normalized vasodilator response, NO bioavailability, and blood pressure. (A) Acetylcholine-induced vasodilatation is greater in IRKO-hIRECO than in IRKO and comparable to that in WT mice (n = 7, 10, and 8, respectively). (B) Acetylcholine EC_50_ data derived from panel (A). (C) SNP-induced endothelium-independent vasodilatation is comparable in IRKO-hIRECO, IRKO, and WT mice (n = 4, 7, and 6, respectively). (D) Insulin-stimulated NO synthase activity is greater in IRKO-hIRECO than in IRKO mice (n = 6, 5, and 6, respectively). (E) Superoxide abundance was similar in IRKO-hIRECO and IRKO mice but greater than in WT mice (n = 6, 7, and 6, respectively). (F) PE-induced vasoconstriction is lower in IRKO-hIRECO than in IRKO mice and comparable in WT mice (n = 7, 10, and 8, respectively). (G) PE EC_50_ data derived from panel (F). (H) Diminished PE-induced vasoconstriction in IRKO-hIRECO mice is caused by increased NO bioavailability as it is significantly abrogated by the NO synthase antagonist L-NMMA (n = 5 and 5, respectively). (I) Systolic blood pressure is lower in IRKO-hIRECO than in IRKO mice and comparable in WT mice (n = 10, 19, and 18, respectively). Sample sizes are presented in the order WT, IRKO, and IRKO-hIRECO. *P < 0.05. EC_50_, half-maximal effective concentration; n.s., nonsignificant for IRKO vs IRKO-hIRECO.

IRKO mice are known to exhibit endothelial dysfunction (3), so next we characterized aortic vasomotion. IRKO-hIRECO mice showed significantly greater endothelium-dependent relaxation in response to acetylcholine than IRKO mice (Fig. 3A and 3B) but identical endothelium-independent responses to SNP (Fig. 3C). Moreover, an *ex vivo* NO synthase activity assay showed increased insulin-stimulated NO production in IRKO-hIRECO vs IRKO mice (Fig. 3D); importantly, there was no difference between WT and IRKO-hIRECO mice, and IRKO mice were impaired compared with WT mice (*P* < 0.05). We have previously demonstrated oxidative stress due to increased superoxide abundance in 6-month-old IRKO mice (6); however, superoxide abundance was similar in 4- to 6-week-old IRKO and IRKO-hIRECO PECs (Fig. 3E). IRKO mice displayed increased vasoconstriction in response to phenylephrine, which was completely normalized (not statistically different from WT levels) in IRKO-hIRECO mice (Fig. 3F and 3G). Furthermore, the normalized contractile response to phenylephrine in IRKO-hIRECO was completely lost in the presence of the NO synthase inhibitor L-NMMA (Fig. 3H), suggesting increased basal vascular NO production. Systolic blood pressure measurement provided *in vivo* corroboration of these data, with that of IRKO-hIRECO mice being lower than that of IRKO and statistically comparable to WT mice [IRKO-hIRECO mean 97.3 (SEM 2.5) mm Hg, IRKO 105.9 (3.0) mm Hg, WT 94.8 (3.0) mm Hg; Fig. 3I].

### IRKO-hIRECO mice exhibit rescue of re-endothelialization after arterial injury

**Figure 4.**
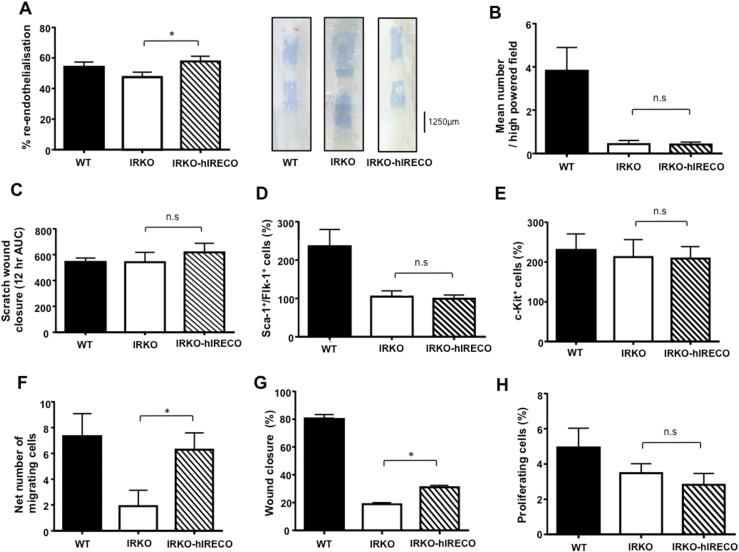
Endothelial repair is normalized in IRKO-hIRECO mice and is associated with increased endothelial cell migration. (A) Re-endothelialization of wire-injured femoral artery is greater in IRKO-hIRECO than in IRKO mice and comparable in WT mice (n = 6, 14, and 12, respectively); representative images are presented adjacent. (B) *In vitro* myeloid angiogenic cell formation was comparable between blood from IRKO and IRKO-hIRECO mice, both of which were lower than in WT mice (n = 5, 5, and 6, respectively). (C) Conditioned medium from IRKO-hIRECO and IRKO spleen-derived myeloid angiogenic cells was equally effective in promoting endothelial scratch wound closure (n = 4, 4, and 4, respectively). No differences were noted in the abundance of (D) circulating Sca-1^+^/Flk-1^+^ or (E) c-Kit^+^ progenitor cells between IRKO-hIRECO and IRKO mice, although the former were lower in IRKO-hIRECO and IRKO than WT mice (n = 7, 8, and 9, respectively). (F) PEC migration toward VEGF-A_165_ in a Boyden chamber apparatus is greater in IRKO-hIRECO than in IRKO mice and comparable in WT mice (n = 9, 9, and 9, respectively). (G) Closure of a scratch wound formed in PEC was also greater in IRKO-hIRECO than IRKO mice, although both were lower than in WT mice (n = 6, 9, and 6, respectively). (H) No difference in PEC proliferation was noted between IRKO-hIRECO and IRKO mice (n = 5, 6, 5, respectively). Sample sizes are presented in the order WT, IRKO, and IRKO-hIRECO. *P < 0.05. AUC, area under the curve; n.s., nonsignificant for IRKO vs IRKO-hIRECO.

Re-endothelialization of the injured femoral artery is also known to be impaired in IRKO mice (4). IRKO-hIRECO mice showed augmented re-endothelialization compared with IRKO mice, which were statistically comparable to WT mice (Fig. 4A). Notably, this finding was not associated with differential *in vitro* formation of myeloid angiogenic cells between IRKO and IRKO-hIRECO blood (Fig. 4B) or in the capacity of conditioned medium from these cells to promote endothelial scratch wound closure (Fig. 4C). Moreover, we noted no differences between IRKO and IRKO-hIRECO mice in the abundance of circulating Sca1^+^/Flk1^+^ (Fig. 4D) or c-Kit^+^ (Fig. 4E) progenitor cells, which have previously been shown to be less abundant in IRKO than in WT mice (4). These data suggest that increased vascular repair in IRKO-hIRECO mice may not be related to changes in circulating proreparative cells. Importantly, migration of IRKO-hIRECO PECs toward VEGF was increased in a Boyden chamber apparatus (Fig. 4F), being statistically comparable to that of WT mice. Moreover, PEC scratch wound closure was significantly greater in IRKO-hIRECO mice (Fig. 4G), although it remained less than in WT mice (*P* < 0.05), and this difference was not associated with any change in PEC proliferation defined by EdU incorporation (Fig. 4H), suggesting that the increased migration noted earlier may underlie improved scratch wound closure.

## Discussion

We have evaluated restored endothelial insulin receptor expression in the context of systemic insulin receptor haploinsufficiency and demonstrated that it does not affect whole-body glucose metabolism, normalizes blood pressure and macrovascular NO bioavailability, and promotes endothelial repair after denuding arterial injury, potentially via increased endothelial cell migration. By rescuing vascular dysfunction with endothelium-specific restoration of insulin receptor expression, we demonstrated that off-target “compensatory” changes in endothelial gene expression do not account for the vascular dysfunction of IRKO mice. In other words, we have strengthened the evidence base showing that endothelial insulin receptor expression is essential for normal vascular function and repair.

The comparable glucose homeostasis of IRKO and IRKO-hIRECO mice concurs with reports that vascular endothelial insulin receptor knockout mice exhibit preserved insulin sensitivity (27). Moreover, recent elegant imaging studies have shown that skeletal muscle transendothelial insulin transport depends on fluid phase transport and not insulin receptors (28). Although studies of vascular endothelial insulin receptor knockout mice produced with a different Cre line have shown slowed insulin signaling kinetics in a tissue-specific manner, these used suprapharmacological insulin doses (>250 times greater than in our insulin tolerance tests) and so are of unclear relevance to physiology (29). Notably, endothelial loss of insulin receptor substrate-2 is also associated with impaired systemic glucose metabolism, which can be rescued by NO donors, reflecting the importance of tissue perfusion to insulin action (30). However, this association does not necessarily imply that endothelial insulin receptors are needed for systemic glucose homeostasis. Although debate is likely to continue, our work and the wider literature suggest that endothelial insulin receptors are not critical effectors of systemic glucose metabolism. This important observation raises the question of why endothelial cells express insulin receptors.

The association between reduced insulin signaling and endothelial dysfunction is well established and is characterized by diminished endothelial NO generation and oxidative stress resulting from increased superoxide production (31–33). Although we noted no difference in endothelial superoxide between IRKO and IRKO-hIRECO mice, this finding may reflect the young age of mice used for endothelial cell isolation in our studies (4 to 6 weeks); notably, we have previously shown greater vascular superoxide abundance in 6-month-old but not 2-month-old IRKO mice (6). Mechanistically, excessive superoxide is an important mediator of the vascular disease associated with insulin resistance, for example by diminishing NO bioavailability and oxidizing lipids, proteins, and DNA (2). Multiple assays in adult IRKO-hIRECO mice showed rescued insulin-induced and isometric tension–induced NO generation, potentially explaining their greater vasomotion and lower blood pressure. Mechanistically, NO has also been proposed to contribute to normal vascular homeostasis by suppressing leukocyte adhesion molecule expression, impeding platelet activation, and retarding vascular smooth muscle proliferation, among many other effects (34). Interestingly, we observed lower phosphorylation of Akt and eNOS in freshly isolated endothelial cells from IRKO-hIRECO (and WT) mice vs IRKO mice, potentially suggesting compensatory *in vivo* adaptations to maintain NO production in IRKO mice. Furthermore, we saw no differences in serum-free and insulin-stimulated Akt phosphorylation of culture-expanded endothelial cells from IRKO, IRKO-hIRECO, and WT mice. This finding may suggest that differences in signaling related to altered receptor expression occur in a narrow range of ligand concentrations, but it is also possible that the receptor has ligand-independent roles, as is well established for other receptor tyrosine kinases (35).

Our data also provide evidence that insulin receptor expression in the endothelium *per se* is essential for normal vascular repair, advancing our observations in mice with whole body insulin receptor haploinsufficiency (4). Although our previous work suggested that altered circulating proreparative cells may diminish vascular repair in IRKO vs WT mice, we found no rescue of these parameters in IRKO-hIRECO mice; however, we did observe a clear improvement in endothelial cell migration. Insulin is an established inducer of endothelial cell migration *in vitro* (36–39), with signaling via PI3-kinase to the cytoskeletal regulator Rac1 often being implicated. Furthermore, insulin has been shown to promote local ATP production during the cell membrane remodeling necessary for migration, by mobilizing glycolytic enzymes from the actin cytoskeleton (36). Therefore, the differences between IRKO and IRKO-hIRECO endothelial cell migration are plausible, and this phenomenon is key to the re-endothelialization of denuded arteries *in vivo* (40). Our findings advance the literature linking insulin’s cognate receptor to *in vivo* vascular repair by suggesting an endothelial cell autonomous contribution, rather than effects solely in other cell lineages participating in tissue repair.

Finally, it is important to contrast the adverse vascular phenotype we have recently reported in hIRECO mice (7) with the normalized vascular function of IRKO-hIRECO mice presented in this article. Both models have unperturbed glucose tolerance and insulin sensitivity, further emphasizing the disconnect between the metabolic and vascular consequences of endothelial insulin receptor manipulation. These data, together with the wider literature discussed earlier, indicate that modest perturbations of endothelial insulin receptor expression or signaling have important consequences for vascular physiology.

It is also important to acknowledge some limitations of our work. First, this study intended to dissect the role of endothelial insulin receptors in normal physiology rather than model the complex multilevel signaling abnormalities of insulin resistance associated with obesity and type 2 diabetes. We therefore do not extrapolate our findings to human disease, or even other murine models of diabetes, and it will be important for future research to extend our work in these models. Second, it is likely that the improvements in vascular function and repair are mediated by complex mechanisms, as supported by the unexpectedly increased Akt and eNOS phosphorylation in freshly isolated IRKO endothelial cells. Moreover, it is likely that expression of the insulin receptor varies among endothelial cells, reflecting their established structural and functional heterogeneity within and between vascular beds. Indeed, recent work suggests greater expression of the insulin receptor in the cerebral vs pulmonary vascular beds, although with broadly stable expression in microvascular and macrovascular endothelial cells within these beds (41, 42). Finally, it is beyond the scope of this study to define detailed mechanistic pathways for restored endothelial function and for vascular repair; future studies will be needed to address both. In particular, these studies will need to further define the molecular perturbations (*e.g.,* to signaling events and local redox milieus) caused by altered endothelial cell insulin receptor expression and to mechanistically link these perturbations to altered cellular and vascular behavior by studying multiple vascular beds.

In conclusion, we provide evidence that restoration of endothelial insulin receptor expression alone is sufficient to prevent the vascular dysfunction caused by systemically reduced insulin signaling. This study emphasizes the pivotal importance of the endothelium as a target of insulin during normal vascular physiology.

## Supplementary Material

Supplemental Figure 1

## Abbreviations:

EdU: 5-ethynyl-2'-deoxyuridine
eNOS: endothelial nitric oxide synthase
hIRECO: human insulin receptor endothelial cell–specific overexpression
IRKO: insulin receptor knockout
NO: nitric oxide
PE: phenylephrine
PEC: pulmonary endothelial cell
SNP: sodium nitroprusside
VEGF: vascular endothelial growth factor
WT: wild-type
