# Supplementary material for: Endothelial Insulin Receptor Restoration Rescues Vascular Function in Male Insulin Receptor Haploinsufficient Mice
Source: Endocrinology. 2018 May 15;159(8):2917–25. doi: 10.1210/en.2018-00215 (PMC6047419; doi:10.1210/en.2018-00215)
Supplement: Supplemental Figure 1 [file en.2018-00215.sf1.pdf]

Supplemental Figure 1: Endothelial insulin signaling is unchanged in IRKO and IRKO-hIRECO.

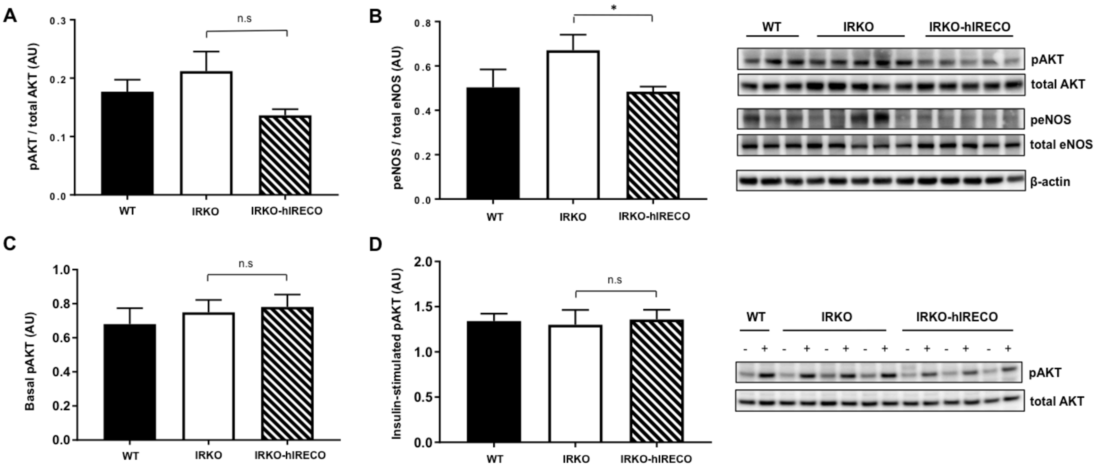

A) Phosphorylated Akt (S473) normalized to total Akt is lower (p=0.06) in freshly isolated non-cultured pulmonary endothelial cells from IRKO-hIRECO than IRKO, and is comparable to wild-type littermates (n=3,5,5); representative immuno-blots are presented to the right of panel B. **B**) Phosphorylated eNOS (S1177) normalized to total eNOS is lower in freshly isolated non-cultured pulmonary endothelial cells from IRKO-hIRECO than IRKO, and is comparable to wild-type littermates (n=3,5,5); representative immuno-blots are presented to the right of the panel. **C)** Phosphorylated Akt (S473) normalized to total Akt is comparable in serum deprived culture-expanded pulmonary endothelial cells from IRKO-hIRECO, IRKO and WT littermates (n=4,6,4). **D)** Phosphorylated Akt (S473) normalized to total Akt is comparable in culture-expanded pulmonary endothelial cells from IRKO-hIRECO, IRKO and WT littermates 15 minutes after exposure to 150nM insulin (n=4,6,4). Sample sizes are presented in the order WT, IRKO, IRKO-hIRECO. * denotes p<0.05.
